# Understanding the structure–function relationship in selected dairy food matrices using material science approaches*

**DOI:** 10.3168/jdsc.2024-0685

**Published:** 2025-03-03

**Authors:** Prateek Sharma, Pragya Choudhary

**Affiliations:** Department of Nutrition, Dietetics, and Food Sciences, Utah State University, Logan, UT 84322

## Abstract

Dairy foods are available in a variety of physical forms, including liquid, semi-solids, and solids. The desired functionality of these foods depends upon the end user application (e.g., viscoelasticity of cheese in high-speed processing, powder rheology for bulk handling, and gelling behavior for texturization). The theoretical basis for functionality in each food matrix is derived from the multiple layers of food structure and the way different molecules interact with one another at different length scales. Modifying food structure through changing formulation or processing conditions is an effective way of controlling functionality. The accurate characterization of food structure and material properties plays a significant role in optimizing functionality and designing equipment. This work presents how the use of novel material science approaches can not only be helpful in understanding the structure–function relationship in various dairy food matrices (slicing of cheese, powder rheology, high-solid spray drying, and cold gelling behavior of highly concentrated micellar casein concentrate), but also in potentially improving the profitability and efficiency of commercial dairy manufacturing operations.

## Highlights

• Dairy foods are available in a variety of physical forms (i.e., liquid, semi-solids, and powders).
• Changing formulation or processing conditions is an effective way of controlling functionality.
• Material science approaches help to understand the structure-function relationships in foods.

## Graphical Abstract

**Figure:**
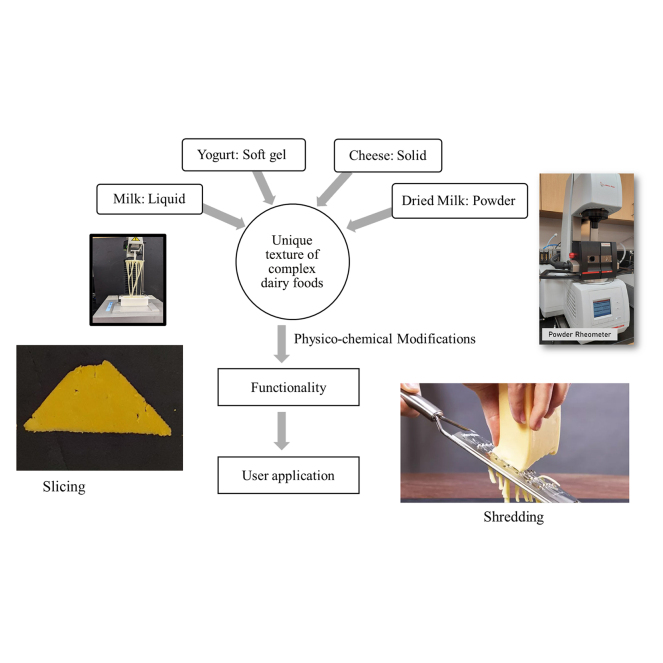
**Summary:** Understanding the structure-function relationship in various dairy food matrices is important for improving the profitability and efficiency of commercial dairy manufacturing operations. Composition and processing conditions of food matrices determine the functionality of dairy foods. Based on the desired functionality of the food, material properties can be used to understand the arrangement of structure at various length scales. Precise measurement of food structure and material properties is important in optimizing functionality through optimum design of equipment or processes. In this work, we have tried to show that the use of novel material science approaches can not only be helpful in understanding the structure-function relationship in various dairy food matrices (slicing of cheese, powder rheology, high-solid spray drying, and cold gelling behavior of highly concentrated micellar casein concentrate) but also in potentially improving the profitability and efficiency of commercial dairy manufacturing operations.

Dairy products are produced in multiple structural forms from liquid milk, a colloidal dispersion of fat and protein in serum. Milk is converted into a soft gel (yogurt) upon acid coagulation or into cheese (solid food) upon rennet coagulation (Lamichhane et al., 2021; Sharma, 2022; Figure 1). With the use of thermal evaporation of the aqueous phase, liquid milk can also be converted into concentrated and dried milk products (Sharma et al., 2016). From the structural perspective, all these dairy products are complex in nature and therefore offer diverse compositional, nutritional, functional, and physiological benefits. Multiscale arrangement of fat, protein, carbohydrates, and mineral content provides a theoretical basis for unique material characteristics. The use of advanced material characterization techniques can be used for understanding structural arrangements of these food constituents at different length scales. This also helps in understanding the role of molecular interactions and their manifestation at microscopic and macroscopic levels, either in the form of textural characteristics or functional performance. The review work presented in this manuscript explains how the use of different material science approaches can be helpful in solving dairy industry problems related to waste minimization and improving sustainability by understanding the process, structure, and property relationship in various dairy foods systems (Figure 2).

**Figure 1:**
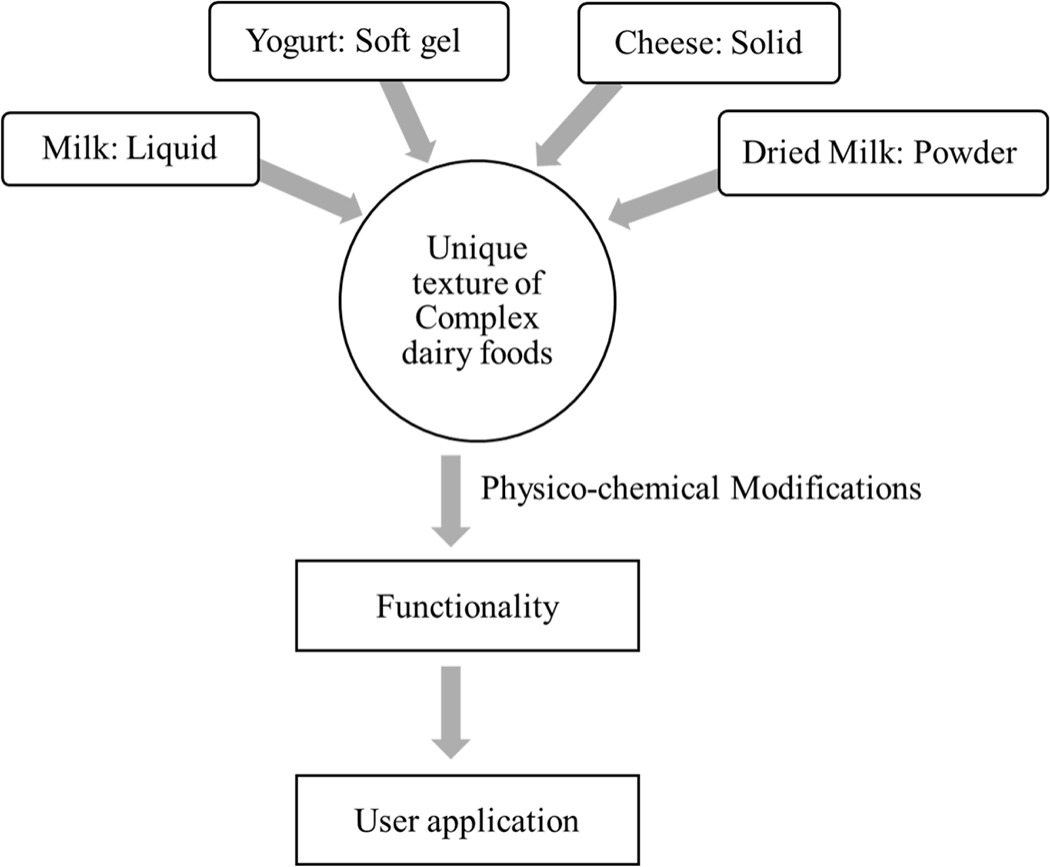
Structure–function relationship in various dairy foods as affected by physical state of the food material and physico-chemical modifications.

**Figure 2:**
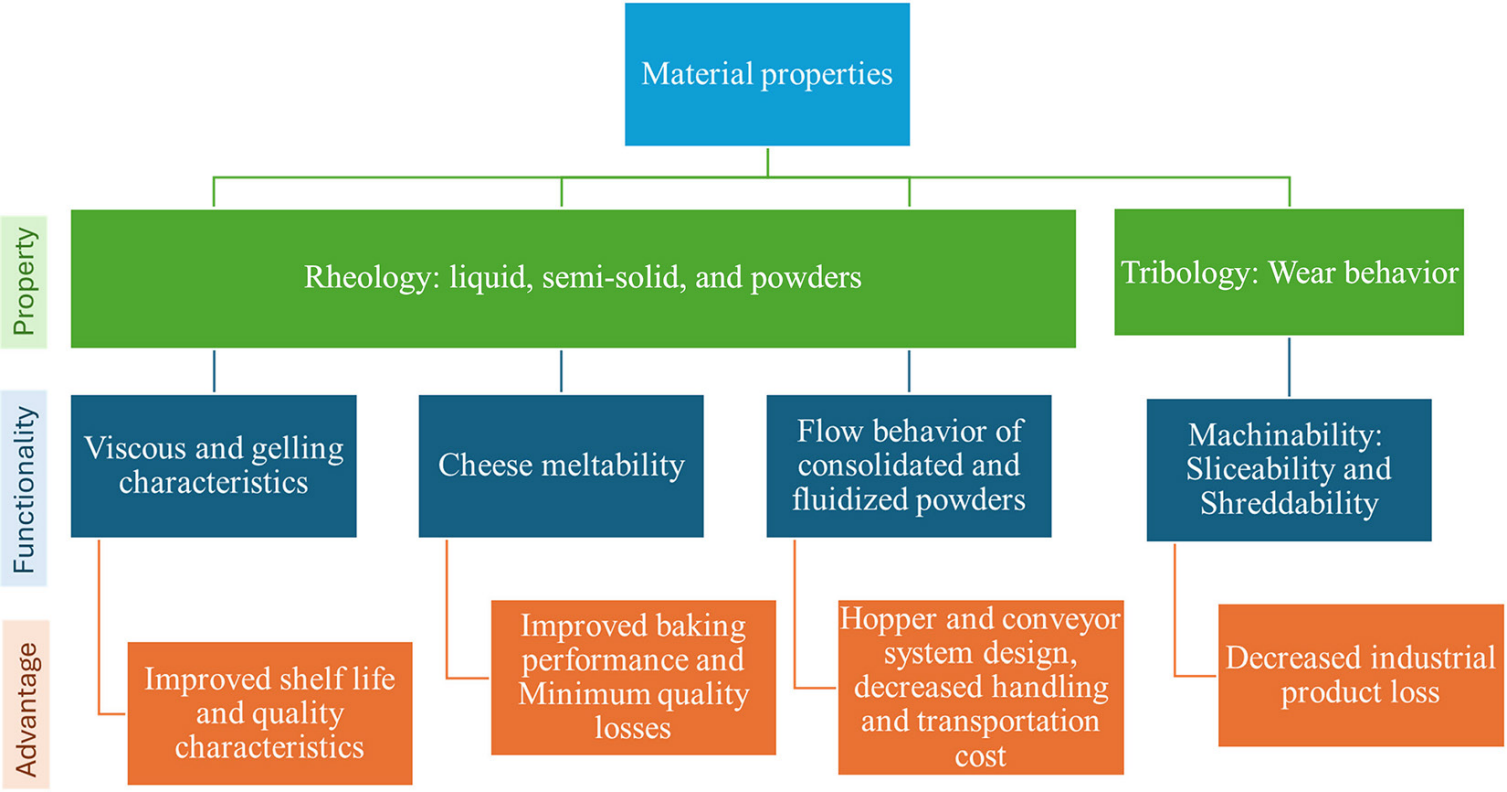
Understanding the role of material properties in optimizing functionality of various dairy foods systems.

Millions of pounds of Cheddar cheese are sold in the United States each year. The cheese industry in the United States is growing at a 4.64% compound annual growth rate (Fortune Business Insights, 2025). In many cases, Cheddar cheese is sliced or shredded on high-speed machines for the use in various applications, such as restaurant services and culinary dishes. Slicing has now become a more common processing operation not only for natural cheeses, but also processed cheeses, both at the manufacturer and consumer end (Apostolopoulos and Marshall, 1994), as it allows faster melting of cheese on a bread roll or burger (Ni and Gunasekaran, 2004). Sliceability is a term that refers to certain physical attributes of the cheese, such as the ease of converting a block into thin slices, resisting breakage or fracture at the slice edge, maintaining the shape and integrity of slices, and undergoing a high level of bending before breaking (Childs et al., 2007; Perrie, 2012; Pace et al., 2024).

On the other hand, shreddability is a comparable property to sliceability that includes several physical attributes, such as the ease of machinability of cheese, the shape and integrity of shreds, the absence of shredding defects such as the propensity of shreds to mat or clump, and the excessive production of fines (Childs et al., 2007). Intuitively, sliceability depends on the chemistry, microstructural, and rheological properties of the casein network. Good shredding or slicing behavior is observed in a relatively narrow range of textural properties and is not fully understood, or controlled, by cheese manufacturers. Soft cheese usually shows poor slicing characteristics because it sticks to the blade and produces slices that tend to stick together or to the moving metal surface. This also causes material loss. On the other end of the spectrum, firm and dry mozzarella or Cheddar cheese easily shatters into fines and fractures upon bending (Kindstedt, 1995; Chen, 2000).

During the process of slicing or shredding, problems such as loss of material can arise from cheese sticking to the moving parts of machinery or crumbling during high-speed operations. This wearing phenomenon or poor performance of cheese during shredding or slicing results in significant revenue losses for cheese manufacturers. Unfortunately, few methods are available to subjectively assess the slicing behavior of cheese (Perrie, 2012; Pace et al., 2024). Currently, we have no objective way to predict attributes of the machinability of Cheddar cheese from which these problems may arise.

Tribology, which is the study of wear and friction behaviors or lubrication between 2 interacting surfaces in relative motion, has emerged as a technique of interest in food research in recent years and has been correlated with various sensory attributes (Nguyen et al., 2016; Laiho et al., 2017; Zhang et al., 2017; Tan and Joyner, 2018). However, despite the fact that this testing mimics the deformation regimen experienced during slicing or shredding operations, there is a lack of research reports regarding its use in determining or predicting the machinability of food materials (e.g., mass loss). In recent years, a study was done on κ-carrageenan and whey protein gels, which introduced a model for the processability of soft materials based on mechanical and wear properties (Tan and Joyner, 2018). The following year, another study demonstrated the correlation between rheological and tribological behaviors of different commercial cheese types. This study correlated penetration depth to firmness and elasticity due to structural differences in the cheese matrices, and large-strain viscoelastic behaviors to wear behaviors (Zad Bagher Seighalani and Joyner, 2019; Zad Bagher Seighalani et al., 2021). There are no reports available in the literature to suggest the correlation between the body and texture of cheese and the wear behavior and its subsequent impact upon machinability, particularly in high-speed processing conditions such as shredding or slicing.

Research is being conducted at Utah State University (**USU**; Logan, UT) to use wear behavior and rheological and textural properties as tools to develop predictive models for the slicing and grating performance of cohort of cheeses (young, medium, and sharp) with varying composition and extent of proteolysis (Pace et al., 2024). These models will allow cheese manufacturers to judge the suitability of the cheese for slicing operations or for use as an ingredient in processed cheese formulation.

A subjective sensory-based method was used by Perrie (2012) for testing sliceability of cheeses. This method was based upon visual analysis of cheese slices cut into a triangle shape using the grading scale developed at USU. Pace et al. (2024) validated a 5-point slice defect score method developed by Perrie (2012) for studying the impact of proteolysis and cheese slice thickness and shape on the slice quality of commercial Cheddar cheese blocks. The study reported that the slice defect score was able to differentiate between different ages, shapes, and thickness of the cheese slices. It was easier to cut 2-mm square slices from the mild cheese blocks. Findings from this work will be used to develop more comprehensive quantitative criteria for defining slicing characteristics. Developing an objective method using wear behavior for shreddability and sliceability (particularly in a similar deformation rate regimen) and establishing its relationship with material characteristics such as elasticity, hardness, adhesiveness, cohesiveness, and wear behavior, would be very helpful in predicting the sliceability or shreddability of cheese at early stages of storage. This will help in the early prediction of sliceability without having to do actual slicing.

Powder rheology involves determining flow behavior of consolidated or fluidized powders. Rheological properties of dairy powders are of great significance in designing equipment for spray drying and fluidized bed drying, and for optimizing parameters for conveying dried product through pipelines and determining optimum conditions for storage and packaging (Palmer et al., 2023). There are numerous challenges in powder processing. Depending upon composition, processing, and storage conditions, powders may tend to cake and clump which causes hindrances during transportation and packaging. Similarly, a caking tendency can affect the rehydration behavior, leading to consumer dissatisfaction. Various factors, including temperature, relative humidity, physical state of the constituents, particle size, surface characteristics, compositional differences, and the duration of storage, can present challenges that might affect the extent of loss of functionality. These challenges include which form of transport is effective for a given powder, what effect will shear rates have on the morphology and structure of powder particle, how much additive is needed to ensure sufficient flow behavior, how much air flow is required to complete fluidization of partially or fully dried powder particle, how to avoid sticking of powder to the sides of drying chamber and product storage silos, how to prevent segregation of powder particles without compromising with flowability, and at what normal force caking of powder will occur during storage of powder. Addressing these challenges will improve the sustainability of dairy powder manufacturing operations by helping minimize product losses during production, transport, and storage. This can be achieved by understanding and controlling critical factors responsible for poor flowability, which necessitates accurate measurement of rheological properties under various stress regimens exerted on powder particles during flowing and rest conditions. Palmer et al. (2023) developed a testing protocol for determining flow function coefficient of dairy powders using shear cell attachment to the strain controlled MCR 302 rheometer (Anton Paar, Graz, Austria). The presence of the stick-slip phenomenon with less cohesive (such as dairy) powders is a common problem that leads to instant flow, absence of failure peaks, or unreliable data. Palmer et al. (2023) overcame the issue of the stick-slip phenomenon using material science approaches for obtaining a reliable flow function coefficient. This testing protocol can now be used to understand the impact of particle size, moisture content and composition on the sticking tendency of dairy protein powders.

Flow properties and structural integrity of dairy powders particles are dependent upon both intrinsic and extrinsic factors. Therefore, flow of powder samples not only depends upon moisture and lactose content, the state of proteins, and the size and density of dried particles, but also on the flow conditions (e.g., airflow pressure, humidity, and temperature). As per our understanding, for a given sample, there must be a critical moisture content below which powder should start fluidizing and becomes easy to transport.

The spray-drying technique is generally employed for drying dairy fluid streams. However, this process is not energy efficient due to the use of hot air as a heating medium, which has poor thermal conductivity. Often, there are heat losses to the atmosphere with the exit air. On the other hand, the use of thermal evaporation under vacuum is a relatively energy efficient process. Therefore, evaporation of water in an evaporator to the maximum extent possible is a good strategy for energy saving and improving sustainability. However, the viscosity of concentrate fluid is a limiting factor for the atomization process used in spray drying. Highly viscous and non-Newtonian liquid materials can lead to clogging of the pressure nozzle or orifice of the centrifugal disk. Filament extension atomization (**FEA**) is a novel material science technique which can be used for atomizing very viscous and sticky fluids at low cost in an energy efficient way (Sangli et al., 2022). This technology uses stretching liquid films (filament) on a counter-rotating drum at a faster rate so that the film or filament breaks into smaller droplets. Droplet breakup dynamics depends upon rheological and surface tension characteristics of the fluid. Therefore, designing processing parameters for FEA requires thorough rheological analysis of high solid loadings of dairy fluids (Zad Bagher Seighalani et al., 2024). Filament extension atomization technology was successfully used for atomizing an 80% solid concentration of reconstituted sweet whey and 50% concentration of whey protein concentrate 80 solids. These concentrations are 30% higher than the current practice in the industry (Sangli et al., 2022).

Demand for high-protein products is on the rise because of their enhanced nutritional and functional benefits. With the invention of a wide range of membrane processing techniques, it is now possible to create different versions of milk protein ingredients, such as micellar casein concentrate (**MCC**), milk protein concentrate or isolate, and whey protein concentrate/isolate and permeate. Highly concentrated MCC (**HC-MCC**) has been used in cheesemaking for standardizing or increasing the protein-to-fat ratio so that cheese yield can be improved (Lu et al., 2016; Xia et al., 2020). However, one of the limitations of the use of this product in cheesemaking or in liquid food applications such as beverages is the formation of cold gels at low temperatures (<10°C; Lu et al., 2015, 2016). Though the exact mechanism of cold gel formation is not yet very well studied, a couple of possibilities have been proposed by researchers. Jamming transition of casein micelles in the dense network could be one of the reasons for this behavior (Lu et al., 2015, 2016). On the other hand, swelling of micelles or overlapping concentrations could also play an important role in the cold gelling behavior (Dunn et al., 2021). The role of calcium also cannot be ruled out, as calcium chelation with the addition of trisodium citrate or calcium ions changes the cold gelling behavior drastically (Pougher et al., 2024). Therefore, common strategies to avoid cold gelling behavior could potentially include holding the product at a higher temperature, dilution, and manipulating the calcium content in the product. To ensure the cold gels are completely melted and well above melting temperature, it is important to accurately measure the cold gelling point of HC-MCC in a way that is independent of experimental conditions. Zad Bagher Seighalani et al. (2021) developed a method for accurately determining the critical gel-sol transition point of HC-MCC by applying the Winter–Chambon criteria (Winter and Chambon, 1986) on multiple waveform rheological data. According to the Winter–Chambon criteria, at the critical state of transition, the loss tangent of a material becomes independent of frequency. The multiwave technique involves the simultaneous application of multiplication oscillation frequencies through stress or strain applications at a given measurement point. The complex signal received because of the multiwave wave application is then filtered through a Fourier transformation to obtain storage and loss modulus (**G′** and **G″**, respectively) at discrete frequencies. The frequency dependence of G′ and G″ with respect to experimental variable (time, temperature, concentration) can then be easily determined without having to run experiments at each frequency. This not only provides better quality data, but also saves a significant amount of experimental time. The findings of this research are now being used for understanding mechanisms of cold gel formation and studying thermoreversibility of these gels.

Overall, determination of the material properties of complex dairy food systems helps to develop new understandings of products, processe,s and prototypes. The optimization of material properties with the use of structure–function relationships can improve profitability and sustainability of dairy processing operations.
